# Neurochemical drivers of effort: The roles of dopamine and beyond in physical and cognitive exertion

**DOI:** 10.1016/j.neubiorev.2026.106696

**Published:** 2026-04-16

**Authors:** Eva Periche-Tomas, Masud Husain, Matthew A.J. Apps, Simon Little, Catherine J. Harmer

**Affiliations:** a Department of Psychiatry, University of Oxford, UK; b Nuffield Department of Clinical Neurosciences, University of Oxford, UK; c Centre for Human Brain Health, School of Psychology, University of Birmingham, UK; d Department of Neurology, University of California, USA

**Keywords:** Effort, Motivation, Dopamine, Apathy, Anhedonia, Neuromodulation

## Abstract

Motivation depends on the ability to evaluate whether potential rewards are worth the effort required to obtain them. Disruptions in this process give rise to debilitating motivational syndromes such as apathy and anhedonia, which are prominent across neurological and psychiatric disorders, including depression and Parkinson’s disease. Although mesolimbic dopamine has long been central to theories of effort-based decision-making, converging evidence indicates that motivational drive emerges from the interaction of dopamine with a broader set of neuromodulatory systems. Here, we review animal and human studies examining the neurochemical regulation of physical and cognitive effort, focusing on dopamine alongside adenosine, GABA, serotonin, norepinephrine, acetylcholine, and metabolic signals. Across paradigms, dopamine plays a key role in energising behaviour and promoting willingness to exert effort, but alterations across both dopaminergic and non-dopaminergic systems can bias cost–benefit evaluations even when reward sensitivity is relatively preserved. These findings help explain why motivational deficits often persist despite dopaminergic treatment and why clinically similar syndromes may arise from distinct underlying mechanisms. By integrating behavioural, pharmacological, computational, and neuroimaging evidence, this review highlights effort-based decision-making as a translatable framework for understanding motivational dysfunction. We argue that progress will depend on moving beyond single-transmitter models toward systems-level approaches that distinguish between different motivational profiles. Such an approach has important implications for both research and clinical practice, offering a basis for more targeted interventions to alleviate apathy and anhedonia and improve quality of life.

## 1 Introduction

Motivation is a fundamental construct in cognitive neuroscience, governing the initiation, persistence, and direction of goal-directed behaviour. It determines how organisms evaluate the trade-off between effort and reward, thereby influencing adaptive decision-making and survival (Salamone et al., 2017). For example, deciding whether to walk up a steep hill for 15 min for a delicious lunch versus walking 15 min along the flat road to the restaurant where the food is the same price but average, requires consideration of the effort as well as the perceived value of the reward.

Motivated behaviour is commonly described as comprising two phases: an appetitive or instrumental phase, involving the allocation of energy and resources to overcome obstacles in pursuit of a goal, and a consummatory phase, in which the goal is achieved or the reward is consumed (McCullough and Salamone, 1992; Salamone and Correa, 2002). The appetitive phase, in particular, highlights the complexity of motivational processes, as it requires dynamic integration between internal states and environmental demands. Importantly, substantial variability exists across individuals and contexts, reflecting situational influences, current requirements and underlying biological mechanisms (Koob, 2010; Salamone et al., 2017).

Motivational impairments lie at the heart of numerous neurological and psychiatric conditions. Apathy and anhedonia are closely related but distinct syndromes. Apathy is typically characterised by reduced motivation and goal-directed behaviour across physical, cognitive, social or emotional domains, whereas anhedonia refers to diminished interest in or pleasure from normally rewarding activities. Although anhedonia can include both experiential (“liking”) and motivational (“wanting”) components, these processes are increasingly recognised as dissociable (Husain and Roiser, 2018). For example, impairments in motivation (e.g., reduced willingness to exert effort) can occur in the absence of deficits in hedonic experience, and vice versa, indicating partially separable underlying mechanisms. Both are debilitating syndromes observed across disorders such as depression (Harmer et al., 2011; Klein, 1974), schizophrenia (Haslam, 1809; Yazbek et al., 2014), Alzheimer’s disease (AD) (Starkstein et al., 2005; Zhao et al., 2016) and Parkinson’s disease (PD) (den Brok et al., 2015; Isella et al., 2003). They have a major impact on daily functioning and wellbeing, yet their underlying neurobiological mechanisms remain incompletely understood. Advancing our knowledge of motivation and reward processes holds promise for developing targeted therapeutic interventions, with the potential to alleviate these symptoms and enhance overall quality of life (Bonnelle et al., 2015, 2016; Chong and Husain, 2016; McCabe et al., 2009; Pulcu et al., 2022; Scholl et al., 2017).

An elusive question has been how neurotransmitter systems, and by implication, drug treatments which target them, regulate behavioural mechanisms such as effort-based choice. Although this remains a topic of ongoing research, Dopamine (DA) has long taken centre stage in this debate, with extensive research in rodent models pointing to the dopamine-rich mesolimbic pathway as a key player in effort-based decision-making for rewards (Husain and Roiser, 2018; Salamone and Correa, 2018). This pathway, originating in the ventral tegmental area (VTA) and projecting to the ventral striatum, particularly the Nucleus Accumbens (NAc) has emerged as a cornerstone of motivated behaviour (Salamone and Correa, 2018). Dopaminergic dysfunction within these pathways has been implicated in motivational impairments observed in disorders such as depression, schizophrenia and PD (Belujon and Grace, 2017; Chanda and Surepalli, 2019; Klein et al., 2019).

However, it has become apparent that effort-based decision-making emerges from a complex interplay of dopaminergic as well as non-dopaminergic systems (Treadway and Salamone, 2022). Exploring this complex modulation of behaviour by different neurotransmitter systems offers an opportunity to uncover more nuanced mechanisms of motivation, paving the way for treatments that extend beyond dopamine to address the broader neurochemical landscape. This review considers neurochemical drivers of effort, focusing on the roles of dopamine and associated modulators in physical and cognitive effort exertion. By bringing evidence from rodent and human studies, we aim to explore the pathways that govern effort-based choice and to inspire new approaches to tackling motivational deficits in clinical settings.

### 1.1 Fundamental Research on Effort-Based Choice

#### 1.1.1 Measuring effort-based decision-making

Effort-based decision-making, a fundamental aspect of motivated behaviour observed across species, involves weighing up different actions associated with different degrees of effort to obtain rewards (Salamone and Correa, 2024). Tasks used to study reward-effort trade-offs often measure how much effort an organism is willing to allocate to obtain a reward, presenting choices between high-effort options leading to highly valued reinforcers and low-effort options yielding lesser rewards (Husain and Roiser, 2018). Common paradigms in animal studies involve lever pressing options that follow either a fixed or progressive ratio schedule, reinforced by highly preferred high-carbohydrate operant pellets. In contrast, the low-effort, low-- preference alternative is standard laboratory chow, freely available in the chamber (Randall et al., 2012; Salamone et al., 1991; Sommer et al., 2014). Similarly, in the T-maze barrier choice task, animals can choose between one arm that requires climbing a vertical barrier to obtain a highly rewarding food and the other arm with no barrier, offering a lower-magnitude reward (Pardo et al., 2012; Salamone et al., 1994; Yohn, Santerre, et al., 2015; Yohn, Thompson, et al., 2015). In humans, physical effort allocation can be adjusted by, for instance, varying the number of button presses, the speed of responses, or the amount of force exerted to obtain rewards. Often, these tasks use choice behaviour as an indication of an individual’s willingness to exert effort (Bonnelle et al., 2015; Chong et al., 2015; Murray et al., 2008; Treadway, Bossaller, et al., 2012). Consistent with this, PET studies have shown that individual differences in striatal dopamine function are associated with variability in effort-based decision-making on tasks such as the EEfRT (Treadway, Buckholtz, et al., 2012). Cognitive effort paradigms have extended this approach to mental demands, requiring participants to choose between low and high-load tasks associated with smaller or larger rewards, thereby capturing the subjective cost of engaging cognitive control (Botvinick and Braver, 2015; Chong et al., 2017; Westbrook et al., 2013; Westbrook and Braver, 2015).

#### 1.1.2 Mesolimbic DA circuitry

##### 1.1.2.1 Animal models

Evidence from rodent models indicates that effort-based decision-making is governed by a distributed forebrain circuitry in which mesolimbic dopamine (DA) plays a pivotal role (Salamone, Correa, et al., 2016; Salamone, Pardo, et al., 2016). Impairments in mesolimbic DA transmission reduce the willingness to exert effort for rewards, without altering primary reward sensitivity. In other words, DA primarily influences the mobilisation of effort to obtain a reward, rather than the hedonic impact of the reward itself (Bailey et al., 2016; Salamone and Correa, 2024).

Early studies using nucleus accumbens (NAc) DA depletions or DA receptor antagonists (D1/D2) revealed consistent “low-effort biases.” Under operant tasks such as fixed-ratio of level presses (FR) or progressive-ratio (PROG)/chow choice paradigms, animals shifted from high-effort lever pressing for preferred food to consuming readily available, less-preferred chow (Aberman and Salamone, 1999; Cousins et al., 1993; Farrar et al., 2007; Randall et al., 2014; Salamone et al., 1991; Yohn, Gogoj, et al., 2016). Comparable effects were found in T-maze barrier tasks, where DA antagonism or accumbens depletion decreased the choice of high-effort, high-reward arms, even though reward discrimination and basic food motivation remained intact (Cousins et al., 1996; Pardo et al., 2012; Salamone et al., 1994; Yohn, Thompson, et al., 2015).

Pharmacological inhibition of DA transmission via tetrabenazine (TBZ), a vesicular monoamine transporter type-2 (VMAT-2) inhibitor, produces a similar low-effort bias in rats and mice across FR, PROG, and T-maze paradigms (Nunes, Randall, Podurgiel, et al., 2013; Randall et al., 2014; Yohn, Santerre, et al., 2015). These effects closely resemble motivational syndromes such as apathy or anergia-like states and are selectively reversed by dopaminergic agents. For example, bupropion and dopamine transport (DAT) inhibitors (e.g., GBR-12909, lisdex-amfetamine, methylphenidate, modafinil) restore normal effort allocation, whereas serotonergic or noradrenergic agents do not (Rotolo et al., 2019, 2020, 2021; Salamone, Yohn, et al., 2016; Yohn, Lopez-Cruz, et al., 2016).

Among dopaminergic agonists, pramipexole (a D2/D3 receptor agonist) has shown particular promise. In rodent models, it reverses motivational deficits induced by 6-hydroxydopamine (6-OHDA) lesions, restoring normal performance on operant and forced-swim tasks without affecting locomotion (Berghauzen-Maciejewska et al., 2014; Favier et al., 2014). Together, these findings indicate that mesolimbic DA transmission is essential for energizing goal-directed behaviour and mediating effort-based decision-making, rather than for encoding primary reward value. Disruptions in this system bias behaviour toward less effortful options, offering a robust translational model for motivational impairments observed in neuropsychiatric disorders.

Although most preclinical work has focused on physical effort, some rodent studies have explored analogous paradigms involving cognitive demand. Tasks that combine reward magnitude with increasing working-memory load have been used to examine whether animals discount rewards when greater mental effort is required. In a direct comparison of physical versus cognitive effort costs, the findings showed that reduced dopaminergic signalling decreased willingness to exert physical, but not cognitive, effort, suggesting that dopaminergic contributions to effort-based choice may be more pronounced for motoric than for mental demands (Hosking et al., 2015).

Other studies have examined dopaminergic modulation of cognitive flexibility and attentional control, showing that striatal and dopamine-dependent prefrontal circuitry dopamine transmission facilitates the ability to update or shift between task rules (Birrell and Brown, 2000; Darvas and Palmiter, 2010; Granon et al., 2000; Ragozzino et al., 1999). The apparent dissociation observed in rodent studies, with dopamine preferentially affecting physical effort, has prompted discussion about potential domain-specific mechanisms. Subsequent analyses suggest that these differences likely reflect methodological and neuroanatomical factors rather than a true dissociation in dopaminergic function. Physical effort tasks are strongly dependent on mesolimbic dopamine transmission within the nucleus accumbens, whereas cognitive effort may rely more on dopaminergic activity in prefrontal and anterior cingulate circuits that were not directly targeted in these experiments (Cools, 2016; Floresco, 2015; Salamone, Correa, et al., 2016; Westbrook and Braver, 2015). Moreover, cognitive-load paradigms in rodents may not fully capture the explicit cost–benefit computations that define cognitive effort, but these findings nevertheless highlight how dopaminergic regulation of effort depends on both task domain and circuitry, contributing to a more nuanced understanding of motivation in animal models.

While extensive animal evidence underscores the role of mesolimbic dopamine in regulating effort-based behaviour (Salamone and Correa, 2012, 2024), other lines of research propose that dopamine may be more closely related to encoding the expected value of future rewards than being primarily sensitive to the effort required to obtain them. From this perspective, dopamine acts as a mediator of reward anticipation and action selection, signalling the motivational significance of potential outcomes rather than simply facilitating motor or cognitive exertion (for a detailed review, see Walton and Bouret, 2019). Consequently, it is important to consider how other neural circuits and neuromodulatory systems interact with dopamine to influence effort-based decision--making. Together, these systems form an integrated motivational network that governs the allocation of effort across both physical and cognitive domains.

#### 1.1.3 Human models

In humans, insights into mesolimbic dopamine function have come largely from studies of Parkinson’s disease (PD), a condition marked by profound dopaminergic degeneration and pronounced motivational deficits. When off dopaminergic medication, PD patients show diminished engagement with effortful actions: they preferentially select low-effort options, exert less force when responding, and are less inclined to participate in tasks that require sustained physical effort (Bouc et al., 2016; Chong et al., 2015; Talbot et al., 2025). These behavioural patterns map closely onto clinical apathy (a common syndrome in PD characterised by reduced initiative, diminished goal-directed behaviour, and blunted emotional engagement). Converging evidence suggests that such apathetic symptoms arise from dysfunction within mesolimbic and frontostriatal dopaminergic circuits, leaving individuals less inclined to mobilise effort or initiate actions in pursuit of rewards (Le Heron et al., 2018).

Evidence examining motivational drive in early-stage Parkinson’s disease (PD) patients, either untreated or off medication at the time of the study, found that these patients applied significantly less grip force than healthy controls, particularly at low incentive levels (Banwinkler et al., 2024). The study also quantified dopamine transporter (DAT) availability in the putamen, showing that the decrease in motor effort was most strongly correlated with a greater loss of putaminal dopaminergic terminals, especially when the incentives were low. The study suggests that putaminal dopamine is crucial for maintaining motor effort, particularly when the perceived reward is small. In other words, patients with lower DAT binding were more significantly affected by low incentive conditions (Banwinkler et al., 2024). This finding aligns with earlier reports that striatal DAT levels are reduced in PD patients experiencing self-reported apathy within two years of diagnosis. However, it contrasts with other evidence that found no significant differences in DAT binding in PD patients with self-rated apathy (Chung et al., 2016). These discrepancies may reflect differences in patient characteristics (e.g., disease stage or severity of apathy), as well as methodological variability in imaging measures and clinical assessments across studies.

In healthy humans, a 2-week administration of the dopamine agonist pramipexole led to enhanced intrinsic motivational vigour, as measured by a rewarded saccade task (Au-Yeung et al., 2024). Additionally, in an instrumental learning reward task, pramipexole improved choice accuracy in the reward condition without affecting performance in loss-related, consistent with computational evidence that it reduces the decay of learned value estimates, thereby preserving reward associations over time (Halahakoon et al., 2024). While not a direct measure of effort-based behaviour, these findings suggest that pramipexole may enhance the stability of reward representations, which could in turn increase the perceived benefits of effortful actions. In clinical research, a preliminary study involving patients with depression and significant anhedonia symptoms, 10 weeks of adjunctive pramipexole treatment (alongside stable doses of antidepressants or mood stabilizers) resulted in significant improvements in striatal reward-related brain activity and reductions in anhedonia scores (Ventorp et al., 2022). Complementing these findings, a large randomised controlled trial in treatment-resistant depression also demonstrated that pramipexole augmentation produced substantial symptom improvement relative to placebo a population typically characterised by marked motivational and reward-processing deficits (Browning et al., 2025). However, these clinical measures do not isolate effort-based or motivational components, and further work is needed to determine the extent to which such improvements reflect changes in effort mobilisation versus other aspects of reward processing.

Beyond physical effort, accumulating evidence indicates that dopaminergic mechanisms similarly govern the motivation to engage in cognitively demanding behaviour. Cognitive effort, defined as the subjective cost of allocating executive control and working-memory resources, has been conceptualised as a decision variable subject to cost–benefit trade-offs analogous to physical effort (Cools, 2016; Westbrook and Braver, 2015). Pharmacological challenge studies in healthy humans have shown that striatal dopamine modulates this computation (Westbrook et al., 2020). Methylphenidate, a dopamine and norepinephrine reuptake inhibitor, increases willingness to engage in cognitively effortful tasks by amplifying the perceived benefits relative to costs of mental work. Similarly, the D_2_ receptor antagonist sulpiride produces comparable effects, most likely by blocking presynaptic autoreceptors and thereby enhancing dopamine release, particularly among individuals with lower baseline striatal dopamine synthesis capacity (Westbrook et al., 2020). These effects have been formalised computationally, showing that dopamine enhances the weighting of benefit information during cost–benefit integration, thereby promoting cognitive motivation.

Further support comes from neuroimaging and clinical data. Dopaminergic medication in Parkinson’s disease reduces avoidance of high-demand cognitive tasks even in the absence of explicit reward incentives (Bogdanov et al., 2022), indicating that dopamine influences cognitive motivation independently of external reinforcement. PET studies have shown that increased cognitive demand elicits dopamine release in prefrontal regions and that greater release is associated with superior task performance (Miederer et al., 2025). Moreover, interindividual differences in dopamine capacity predict the degree of neural adaptation and cognitive performance under high working-memory load (Garrett et al., 2023). Together, these findings suggest that dopamine plays a domain-general role in motivating both physical and cognitive effort by engaging similar processes that evaluate the expected value of actions and amplify the motivational pull or “wanting” of potential rewards within cortico-striatal circuits. In this way, dopamine links cognitive control to broader motivational mechanisms.

### 1.2 Role of Non-Dopaminergic Systems

Increasing evidence points to various interconnected brain circuits playing a role in regulating effort-based choice. These include regions such as the anterior cingulate, prefrontal, insula and orbitofrontal cortices (Domingues et al., 2022; Hart et al., 2017, 2020; Münster, Sommer, et al., 2020; Münster, Votteler, et al., 2020; Walton et al., 2006; Walton et al., 2003) connections between the basolateral amygdala and prefrontal cortex (Floresco and Ghods-Sharifi, 2007); connections between the prefrontal cortex and NAc (Hauber and Sommer, 2009); circuits involving the NAc and ventral pallidum (Farrar et al., 2007; Mingote et al., 2008), NAc and Ventral subiculum (Lindenbach et al., 2022) and NAc and VTA (Ghods-Sharifi and Floresco, 2010; Sokolowski and Salamone, 1998). Besides DA, various neurotransmitters and neuromodulators are involved in the pathways that facilitate effort-based processes.

#### 1.2.1 Adenosine

Substantial evidence indicates that DA interacts with the neuro-modulator adenosine in striatal regions, including the neostriatum and nucleus accumbens (NAc). In the striatum, adenosine A2A receptors are highly expressed and frequently co-localized with DA D2 receptors (DeMet and Chicz-DeMet, 2002; Ferré et al., 2004; Schiffmann et al., 1991). A2A receptor antagonists indirectly enhance DA signalling by lifting the inhibitory influence of A2A receptors on DA release and D2 receptor function, and drugs targeting adenosine receptors can produce behavioural effects. For example, caffeine and other methylxanthines (e.g. theophylline) act as mild stimulants by antagonizing adenosine receptors (Ferre et al., 2008; López-Cruz et al., 2014, 2016). Moreover, A2A receptor antagonists have been studied as potential treatments for Parkinson’s disease (Correa et al., 2004; Pinna et al., 2005; Salamone, Betz, et al., 2008; Salamone, Ishiwari, et al., 2008).

Stimulation of adenosine A2A receptors in the NAc seems to produce effects on effort-related choice similar to those caused by DA depletions or antagonism in the same region. In rats, infusion of the A2A agonist CGS 21680 directly into the NAc altered effort-related choice, reducing Fixed Ratio 5 lever pressing and increasing chow intake (Font et al., 2008). Conversely, control infusions into the overlying dorsomedial neostriatum had no impact. Moreover, rats responding to a Fixed Ratio 10/VI 60 schedule showed reduced lever pressing after intra-accumbens injections of CGS 21680, whereas their performance on a standard VI 60-second schedule (a variable-interval task with no escalating effort requirement) remained unimpaired (Mingote et al., 2008). This suggests that adenosine A2A agonists specifically impair effort-related behaviours, similar to the effects observed with DA depletions and antagonists.

In line with this, evidence has shown that adenosine A2A receptor antagonists, such as MSX-3, can effectively reverse the low-effort bias induced by DA antagonists. For instance, MSX-3 was able to counteract the effects of haloperidol on Fixed Ratio 5 lever-pressing /chow-feeding choice task, restoring normal effort-related responses in rodents (Farrar et al., 2007). Similarly, MSX-3 reversed the suppression of Fixed Ratio 5 lever-pressing caused by the DA D2 receptor antagonist eticlopride, demonstrating its potential efficacy in modulating effort-related choice (Farrar et al., 2010; Worden et al., 2009). In studies involving TBZ, MSX-3 again showed its potential by reversing the decreased lever-pressing (Nunes, Randall, Hart, et al., 2013). Moreover, MSX-3 mitigated the effort-related deficits observed in a progressive ratio (PROG)/chow procedure induced by TBZ, ecopipam and eticlopride (Randall et al., 2014). Additionally, in an effort-based T-maze task, MSX-3 restored the selection of high-effort choices in the presence of TBZ, further highlighting its role in effort-related decision-making processes (Yohn, Thompson, et al., 2015).

Several other adenosine A2A antagonists have also been shown to reverse the low-effort bias induced by DA antagonists using the concurrent lever pressing/chow feeding task paradigm. For example, Lu AA47070 was able to reverse the effects of a low dose of the DA D2 antagonist haloperidol (Collins et al., 2012). A study that systematically compared adenosine A1 and A2A receptor antagonists for their ability to reverse the effects of the DA D1 antagonist ecopipam and the D2 antagonist eticlopride revealed contrasting outcomes. The A1 receptor antagonists DPCPX and CPT failed to reverse the effects of either DA antagonist. In contrast, the A2A antagonist KW-6002 robustly reversed the effects of eticlopride and partially reversed the effects of ecopipam (Nunes et al., 2010). Further evidence found that A2A antagonist MSX-4 reversed the effects of the DA D2 antagonist eticlopride (Santerre et al., 2012). Similarly, KW-6002 and the nonselective adenosine antagonist caffeine significantly reversed the effects of haloperidol, which had reduced lever pressing and increased chow intake (Salamone et al., 2009).

While the role of adenosine receptors, particularly A2A receptors, in effort-related decision-making and motivational processes, has been well-established in animal models, translating these findings to human research presents considerable challenges as well as opportunities. Studies have shown that manipulating adenosine signalling may influence depressive symptoms, with evidence that A2A receptor antagonists could potentially reverse low-effort biases linked to DA dysfunction (Sikka et al., 2022). However, the translation from animal to human conditions remains complex. For instance, a study using PET found that, despite preclinical evidence suggesting that electroconvulsive therapy increases adenosine receptor availability in rodents, this was not the case in patients with MDD (Kroll et al., 2023). As changes in receptor availability in these contexts may not align directly with the behavioural effects seen with acute A2A antagonism in animals, the extent to which preclinical findings translate to humans is still uncertain.

At the same time, emerging work on the broader purinergic system offers complementary mechanistic insights. Recent studies show that ATP released from astrocytes can modulate depressive-like behaviours; after release, ATP is metabolised into adenosine, which then acts on adenosine receptors to influence mood regulation. This line of research underscores the therapeutic potential of targeting adenosine signalling more broadly, beyond receptor-specific pharmacology, to modulate depressive symptoms (Gomes et al., 2021; Sikka et al., 2022). The significance of purinergic signalling, which includes adenosine receptors, as a potential treatment avenue for depression is increasingly recognized, and specific clinical trials in humans are still underway. Overall, while there is significant potential for targeting adenosine receptors in treating depression and motivational deficits, further clinical research is necessary to elucidate the therapeutic implications of these findings and to establish effective treatment protocols based on adenosine modulation.

### 1.3 GABA

Another neurotransmitter linked to effort-based decision-making is gamma-aminobutyric acid (GABA), particularly in the connections between the NAc and the ventral pallidum. Spiny neurons in the NAc, which co-localize DA D1 and D2 receptors as well as adenosine A2A receptors, utilize GABA to project to the ventral pallidum (Salamone et al., 2010).

Several studies have explored the role of GABA in effort-related decision-making using the concurrent lever pressing/chow intake task paradigm. For example, infusions of the GABA agonist muscimol into the ventral pallidum were found to decrease lever pressing for preferred food while increasing consumption of the less preferred chow. Notably, these infusions had no significant effect on food preference in free-feeding choice tests, and injections of muscimol into a control site dorsal to the ventral pallidum did not significantly affect lever pressing or chow intake. These results indicate that stimulation of GABA receptors in the ventral pallidum produces behavioural effects similar to those observed with accumbens DA depletions (Farrar et al., 2008).

Additionally, research has shown that activation of accumbens adenosine A2A receptors increases extracellular GABA levels in the ventral pallidum. In a study using “disconnection” methods (where the A2A receptor agonist CGS 21680 is injected into one hemisphere of the accumbens and the GABA agonist muscimol into the contralateral ventral pallidum) the response output on an instrumental task with high work demands was impaired. This approach selectively disrupted the pathway connecting these two brain regions, highlighting the role of increased transmissions in the ventral pallidum in disrupting effortful task performance (Mingote et al., 2008).

In humans, the role of GABA in effort-based decision-making is complex with evidence showing mixed effects from both GABA agonists and antagonists. As mentioned above, increased GABA transmission has been shown to reduce motivation in animal models. In humans, however, lower GABA levels in regions like the anterior cingulate cortex have been associated with increased apathy and motivational deficits, particularly in disorders like depression (Sarawagi et al., 2021). Interestingly, both GABA agonists and antagonists have demonstrated potential antidepressant effects possibly by modulating brain regions involved in mood and motivation. For instance, GABA-A receptor antagonists may transiently enhance neural plasticity improving motivational and cognitive performance in some contexts (Liwinski et al., 2023; Luscher et al., 2023; Sarawagi et al., 2021). This suggests that GABA’s role in motivational deficits is multifaceted, and the mechanisms in humans may not be entirely parallel to those observed in animal models. More evidence is needed to clarify how GABAergic modulation can hinder or improve motivational impairments, depending on the context and specific brain regions involved, as well as its interaction with other neurotransmitter signals. Understanding these complex interactions may help develop targeted treatments for motivational deficits in mood disorders and other conditions.

#### 1.3.1 Serotonin, Norepinephrine and Acetylcholine

The role of the monoamine serotonin (5-HT) in effort-based choice remains unclear. In rats, some evidence suggests that serotonergic modulation may diminish the willingness to exert effort in effort-based decision-making tasks. For example, administration of the SSRI escitalopram failed to counteract the shift from lever pressing on an Fixed Ratio 5 schedule to chow intake induced by the DA-depleting agent tetrabenazine (TBZ), indicating limited efficacy in promoting effortful behaviour (Yohn, Collins, et al., 2016). Similarly, the SSRI fluoxetine was also unable to reverse TBZ-induced behavioural impairments. This suggests that serotonergic agents may not facilitate (and might even hinder) effort-based decision-making (Yohn, Errante, et al., 2016).

Additional research using the Effort-Related Choice Task (EfRT) demonstrated that acute treatment with conventional antidepressants did not encourage a shift toward high-effort, high-value options. Specifically, citalopram reduced lever pressing, indicating a reduction in effortful responding associated with serotonergic activation (Griesius et al., 2020). Further evidence demonstrated that fluoxetine suppressed high-effort activities such as lever pressing and wheel running in rats (Presby et al., 2021).

A broader analysis of neurotransmitter systems revealed a distinction between the roles of DA and serotonin in decision-making. While DA was crucial for decisions involving both effort and delay in the T-maze task, serotonin appeared to be primarily involved in delay-related decisions rather than those requiring effort. In this context, treatment with haloperidol reduced the willingness to exert effort for higher rewards, whereas pCPA(a chemical compound that reduces serotonin production in the brain by inhibiting tryptophan hydroxylase) increased the preference for immediate, smaller rewards without affecting effortful decisions (Denk et al., 2005). This shift towards immediate gratification in serotonin-depleted rats suggests a link between reduced serotonin levels and heightened impulsivity. The findings align with broader evidence that indicates serotonin plays a role in impulse control, where its depletion may lead to a preference for quick, less effortful rewards (Linnoila et al., 1983; Soubrié, 1986; Winstanley et al., 2005). Additionally, further evidence from a study using pCPA in rats performing the T-maze task revealed dose-dependent effects. Consistent with previous evidence, a standard dose of pCPA did not impact effort discounting, while higher doses impaired the ability to recognise or respond to reward-predictive signals, potentially indicating a reduction in motivation or interest in seeking rewards (Izquierdo et al., 2012).

However, recent studies underscore the significant role of specific serotonergic receptor activity in modulating physical effort and goal-directed motivation. Activation of 5-HT6 receptors in the NAc with the agonist EMD 386088 markedly increased both appetitive and consummatory motivation, as evidenced by enhanced effort in a progressive ratio task and greater food consumption. However, blocking these receptors with the antagonist SB 252585 did not affect motivation (Anderson et al., 2023). Similarly, treatment with the 5-HT2C receptor inverse agonist SB242084 increased instrumental behaviour in various effort-based tasks, suggesting that reducing 5-HT2C receptor activity enhances the willingness to exert effort (Bailey et al., 2016). Conversely, the 5-HT1A receptor agonist 8-OH-DPAT was shown to reduce effort-based decision-making by decreasing serotonin turnover and diminishing rats willingness to exert effort in a T-maze task (Kunčická et al., 2024). These findings collectively highlight the nuanced role of serotonin in regulating physical effort, with potential implications for developing treatments targeting specific 5-HT receptors to enhance motivation in clinical populations.

Human studies have also explored serotonin’s influence on effort choice. SSRIs are widely prescribed as antidepressants, but they have demonstrated limited efficacy in addressing motivational symptoms, particularly those related to behavioural activation and effort exertion, which often remain resistant to treatment (Fava et al., 2014; Padala et al., 2012; Rothschild et al., 2014). However, emerging evidence suggests that serotonin may also influence the cost of both physical and cognitive effort. In a study involving 58 healthy participants treated with either the SSRI escitalopram or a placebo for 8 weeks, participants on escitalopram were more willing to exert physical effort in a handgrip task to obtain monetary rewards, ultimately earning higher rewards. Computational modelling revealed that this effect was driven by a reduction in effort cost rather than changes in reward sensitivity or the valuation of monetary incentives (Meyniel et al., 2016). Similarly, a 7-day course of citalopram in healthy individuals performing a cognitive effort task (sequential information gathering task) found that participants on citalopram were more willing to exert cognitive effort, an effect linked to a reduction in the aversive cost of deliberation, as identified through computational modelling (Michely et al., 2023).

In PD patients, administration of citalopram over 7 days revealed that it increased willingness to exert physical effort for rewards, an effect that was modulated by baseline apathy. Specifically, citalopram increased effort in motivated patients while decreasing it in those with higher levels of apathy, further suggesting a modulatory role for serotonin in effort-based decision-making, which may interact with other factors (Nobis et al., 2023). These findings indicate that serotonin may contribute to the physical and cognitive costs associated with motivation, though further evidence is required to determine its relevance for therapeutic approaches targeting motivational deficits in psychopathology. In line with this complexity, serotonergic antidepressants have also been associated with the emergence of apathy or reduced motivation in some patients, often referred to as SSRI-induced apathy syndrome (Barnhart et al., 2004; Padala et al., 2012). This phenomenon is characterised by diminished initiative and emotional blunting, and may reflect serotonergic modulation of motivational processes that is dissociable from effects on mood as well as broader downstream interactions with other neurotransmitter systems.

Regarding norepinephrine (NE), one theoretical framework suggests that it plays a crucial role in effort by coactivating the autonomic system and the locus coeruleus (LC), the primary noradrenergic nucleus, in response to upcoming challenges. This coactivation enables the organism to mobilise both physical (muscular) and physiological (autonomic) energy to overcome the difficulty. Within this framework, the noradrenergic system is primarily involved in processing effort as a cost signal. It encodes information about upcoming demands and energises behaviour by mobilising physiological and cognitive resources required for action initiation and maintenance, thereby influencing the decision to engage with a challenge. In contrast, DA is thought to primarily encode the expected value of potential rewards, signalling the benefits associated with goal attainment. (Bouret et al., 2012; Sara and Bouret, 2012).

In non-human primates, the activity of DA neurons in the substantia nigra and NE neurons in the LC was compared during a reward/effort trade-off task in rhesus monkeys. The results demonstrated that DA neurons encoded information about expected rewards and were less sensitive to upcoming effort expenditure. In contrast, NE neuron activity increased with both pupil dilation and effort production, suggesting a role for NE in energising behaviour to meet challenges (Varazzani et al., 2015). Consistent with this, rhesus monkeys performing a task that balanced a reward (e.g., juice) against grip force exertion found that systemic clonidine injections, which lower NE transmission, led to reduced force exertion without affecting reward sensitivity (Borderies et al., 2020; Jahn et al., 2017). In rats, however, NE facilitation with atomoxetine, a selective norepinephrine reuptake inhibitor, did not result in increased level pressing on the PROG/chow intake schedule (Yohn, Errante, et al., 2016). Comparable outcomes using atomoxetine were observed in rats subjected to an effort-discounting task (Hosking et al., 2015).

The mixed findings across these studies highlight the complexity of understanding the distinct and overlapping roles of NE and DA in effort-related behaviours. For instance, clonidine also reduces dopamine levels in the medial prefrontal cortex, as noradrenergic neurons in this region are a significant source of dopamine. Therefore, it remains unclear whether clonidine’s main effect on effort may be due to decreased NE or DA transmission (Devoto et al., 2019, 2020).

A complementary line of research suggests that NE contribute more broadly to functions that support effort exertion, particularly attention, cognitive control and persistence, while DA primarily regulates behavioural activation and vigour. This was proposed in a study where the authors compared the effects of chemogenetic inhibition of LC NE neurons and VTA DA neurons in mice during a five-choice serial reaction time task aimed at assessing attention and response control. Inhibition of DA neurons led to significant impairments in behavioural activation and response vigour, suggesting a role for DA in energising behaviour. In contrast, inhibiting NE neurons reduced attentional performance, especially under variable and demanding conditions (Fitzpatrick et al., 2019).

In humans, NE also appears to modulate effort exertion and motivational drive. For instance, atomoxetine has been shown to improve the regulation of alertness and the ability to sustain effort in adults with ADHD (Brown et al., 2011). Amphetamines, like D-amphetamine, have indeed been found to improve effort-based decision-making in tasks requiring healthy participants to exert high effort for high rewards (Soder et al., 2021), although their effects are often interpreted within a dopaminergic framework. However, because amphetamines act on both NE and DA systems, isolating the precise contribution of each neuro-transmitter remains challenging. While DA primarily seems to enhance reward value and influence motivation, NE may play a greater role in regulating arousal, attention, and cognitive control processes that support sustained effort. Yet, their contributions are deeply interconnected, reflecting the joint regulation of motivation, vigour, and attentional engagement across overlapping catecholaminergic circuits.

Another neurotransmitter present in the entire striatal complex, including the NAc, is acetylcholine (Ach) (Nunes et al., 2022; Pisani et al., 2007). Within the NAc, cholinergic interneurons release Ach locally, which acts on muscarinic and nicotinic Ach receptors. These receptor classes exert opposing influences on dopamine transmission. Nicotinic receptors on DA terminals facilitate release, whereas muscarinic receptor activation can inhibit it, leading to complex and context-dependent effects on motivated behaviour (Collins et al., 2019, 2012; Threlfell and Cragg, 2011; Yorgason et al., 2017). Local injections of the muscarinic agonist pilocarpine directly into the NAc core caused changes in effort-related choice behaviour similar to those caused by DA antagonism or A2A receptor stimulation, which resulted in decreased lever pressing but increased chow intake in rats performing a concurrent fixed ratio/chow feeding choice task (Nunes, Randall, Podurgiel, et al., 2013). In humans, healthy participants under the influence of muscarinic 1 receptor antagonist biperiden exhibited an increased willingness to exert physical effort on an effort-discounting task (Abdoust et al., 2024). In non-human primates, striatal cholinergic interneurons have been shown to increase their activity during trials requiring high effort or yielding small rewards, suggesting that Ach signalling tracks effort-related costs and may play a conserved role in modulating cost-–benefit decisions across species (Nougaret and Ravel, 2015).

Other brain modulators have also been shown to influence effort-based choice. Higher levels of glutathione (GSH), an important brain antioxidant, in the NAc strongly predicted greater persistence and sustained effort expenditure over time in effort-related tasks in both humans and rats (Zalachoras et al., 2022). Orexinergic receptor 1 (involved in the regulation of arousal and wakefulness) in the NAc was able to reverse the low-effort preference induced by the dopamine antagonist Olanzapine in rats performing the T-maze barrier task (Nasrollahi et al., 2021). Additionally, in rats performing a PROG ratio task, the genetic deletion of the neuronal calcium sensor-1 (which diminishes DA transmission in the NAc), led to reduced motivation to work for food (Ng et al., 2016).

In addition to these modulators, emerging MRS studies suggest that metabolite balance itself may shape effort allocation. For instance, prolonged engagement in cognitively demanding tasks was shown to increase glutamate levels in the lateral prefrontal cortex, which coincided with greater subjective fatigue and a preference for low-effort options (Wiehler et al., 2022). Ultra-high-field ^1H-MRS in the NAc further revealed that individual differences in the glutamine-to-glutamate ratio predict motivated performance, with higher ratios linked to greater stamina and reduced perceived effort, while social competition enhanced performance particularly in individuals with lower ratios (Strasser et al., 2020). Functional MRS (fMRS) studies also demonstrate dynamic metabolic shifts during sustained motor activity, such as task-related increases in lactate and glutamate in the motor cortex (Morelli et al., 2025).

**Fig. 1:**
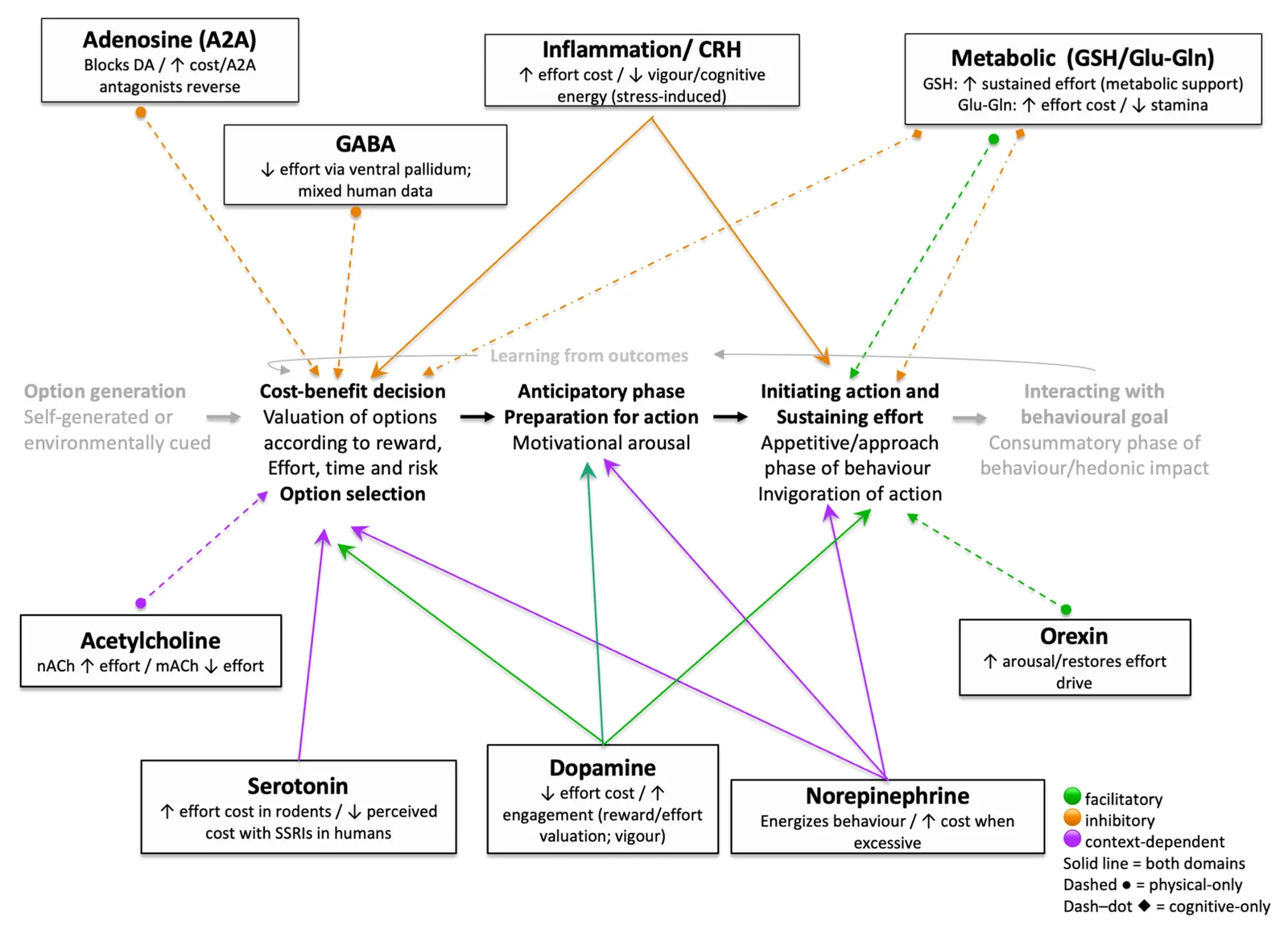
Integrated neuromodulatory influences across the behavioural components of effort-based decision-making. This schematic integrates evidence from animal and human studies to illustrate how different neuromodulatory systems shape some of the major stages of effort-based decision-making. Bubble colours denote the predominant direction of influence: green for *facilitatory* effects (reducing effort cost or enhancing engagement), orange for *inhibitory* effects (increasing perceived effort cost, reducing vigour or stamina), and purple for *context-dependent* or receptor-specific actions.Arrow styles indicate the *domain* of modulation.Solid arrows represent modulatory influences supported in *both physical and cognitive* effort paradigms. Dashed arrows with a circular tail denote influences observed *only in physical-effort* paradigms. Dash–dot arrows with a diamond tail denote influences observed *only in cognitive-effort* paradigms.Arrows originating from each neuro-modulator point toward the behavioural components for which evidence exists (such as cost–benefit valuation, preparation for action, or the initiation and maintenance of effort). Components with no confirmed modulation in the reviewed literature (e.g., option generation or consummatory/hedonic phases) are shown without incoming arrows. This figure highlights how diverse neuromodulatory systems converge on specific stages of effort allocation, with dopaminergic and noradrenergic pathways influencing multiple components, while others, such as adenosine, GABA, or metabolic fatigue signals, act more selectively. The behavioural-component structure is adapted from Husain and Roiser (2018).

Altogether, this evidence underscores that effort-based choice arises from the interplay of diverse neurotransmitter systems, neuro-modulators, and metabolic processes, highlighting the need for future work to clarify how these elements converge to shape motivated behaviour. This interplay is summarised in Fig. 1, which maps the modulatory influences of each system onto the key behavioural components of effort-based decision-making. To complement this schematic overview, Table 1 summarises studies across neurotransmitter systems, highlighting species, effort domain and key behavioural effects.

## 2 Effort-based decision-making and motivational impairments in depression and stress-related disorders

Evidence shows that depression-related conditions trigger a low-effort bias in rodents during effort-based choice tasks, underscoring the relevance of these paradigms for modelling the motivational impairments central to depression, Parkinson’s disease, and schizophrenia. For instance, stress has been shown to shift rodents towards low-effort choices, a finding that parallels the association between chronic stress and mood disorders in humans as well as the link to maladaptive reward and motivation behaviours in animals. Acute stress, such as 1 h of restraint, decreased preference for more costly rewards and increased decision latencies, suggesting a selective interference with cost/benefit evaluations related to effort (Shafiei et al., 2012). Similarly, chronic social defeat stress reduced motivation for lever pressing in a progressive ratio task and shifted behaviour towards lower, easier rewards (Dieterich, Yohn, et al., 2020; Dieterich et al., 2021), indicating how chronic stress may induce maladaptive shifts in effort-related decision-making and diminish motivated behaviour.

Corticotropin-releasing hormone (CRH) plays a crucial role in this stress-related modulation of effort-based decision-making. For example, chronic administration of corticosterone (CORT) in rats led to reduced lever pressing in sessions where lab chow was concurrently available and decreased high-reward arm choices in a Y-maze when more effort was required, shifting preference to a lower reward option (Dieterich, Stech, et al., 2020). These findings demonstrate that chronic CORT reduces the motivation to work for a highly rewarding reinforcer when a less demanding option is available. Similarly, central infusion of CRH mimics the effects of stress by increasing decision latencies and reducing response vigour without altering preference for larger versus smaller rewards. CRH infusions reduced response for food delivered on a progressive ratio, suggesting an amplification of perceived effort costs required to obtain rewards (Bryce and Floresco, 2016). These findings underscore the involvement of CRH in regulating effort-related decisions and suggest that heightened CRH activity may contribute to the motivational impairments and abnormal decision-making associated with stress-related psychiatric disorders such as depression (Hupalo et al., 2019).

In humans, research has further validated the relationship between low-effort biases and motivational dysfunction in depression. For example, investigations deploying effort tasks such as the Apple Gathering Task or the Expenditure for Rewards Task (EEfRT) have reported that participants with depressive symptoms tend to select low-effort options even when greater rewards are at stake, indicating a diminished willingness to expend effort for higher rewards (Treadway, Bossaller, et al., 2012; Treadway and Zald, 2011; Valton et al., 2024). Studies involving physical effort production have demonstrated that people with depression tend to exhibit a lower grip in response to motivational cues, alongside rating effort tasks as more demanding, which highlights a perceived difficulty in effortful tasks (Ganipineni et al., 2023). This behaviour aligns with the reduced drive commonly associated with depression, particularly in relation to anhedonia, where patients may experience reduced pleasure, as well as reduced motivation to engage in rewarding activities.

Additional evidence from cognitive effort tasks, such as the Cognitive Effort Discounting Task (CEMT), reinforces these findings, showing that individuals with MDD were often less willing to exert effort for reward compared to their healthy counterparts, frequently choosing low-effort, low-reward options (Ang et al., 2023). With regard to the neural correlates, neuroimaging studies have revealed abnormalities in the frontostriatal circuitry associated with depression. In particular, depressed patients often show altered activation and connectivity across key regions implicated in reward processing, including the striatum, ventromedial and medial prefrontal cortices (vmPFC, mPFC), dorsolateral prefrontal cortex (dlPFC), and subdivisions of the anterior cingulate cortex—perigenual (pgACC), subgenual (sgACC), and dorsal (dACC) areas. These regions collectively contribute to evaluating reward magnitude, predicting effort costs, and integrating motivational value signals to guide behaviour. Disruptions within this network are thought to underlie the blunted reward responsiveness and reduced willingness to exert effort characteristic of depression (Pizzagalli, 2022).

Other lines of research highlight significant crosstalk between cen-tral neurotransmission and peripheral inflammatory responses in relation to motivational symptoms. Growing evidence points to the role of inflammation and the innate immune system in the pathophysiology of neuropsychiatric disorders such as depression, Parkinson’s disease, and schizophrenia. In particular, cytokine responses have been associated with motivational deficits like fatigue or anergia (Dantzer, 2009; Dantzer et al., 2008, 2012; Miller & Timmie, 2009). In patients treated for Hepatitis C, the effects of chronic administration of IFN-α have been associated with the subsequent development of depressive episodes in one-third of the treated patients (Capuron et al., 2002; Udina et al., 2012), with higher occurrence of symptoms such as lack of energy, fatigue or psychomotor retardation (Capuron et al., 2007; Dowell et al., 2016, 2019; Felger et al., 2012; Felger and Treadway, 2017).

Experimental studies in healthy humans have also demonstrated that various immune challenges such as lipopolysaccharide (LPS), vaccines and pro-inflammatory cytokines can quickly induce disturbances in mood, motivation and cognition that have remarkable similarities to the symptoms of depression (Dantzer et al., 2008; Harrison et al., 2009, 2015, 2016). In rats, the cytokines IL-1β and IL-6 induced a preference for low-effort tasks in the Fixed Ratio 5/chow-feeding schedule. This low-effort bias was reversed by dopamine transporter inhibitors like methylphenidate and lisdexamfetamine, as well as the adenosine A2 antagonist MSX-3 (Nunes et al., 2014; Yohn, Arif, et al., 2016). In humans, experimental models of endotoxemia using LPS have yielded inconsistent findings. LPS can reduce the selection of high-effort trials in an effort-stake task (Draper et al., 2018). However, contrasting results were observed in the **EEfRT** task, where LPS administration in healthy participants enhanced performance in high-effort/high-reward trials (Lasselin et al., 2017). These findings point to a crucial interaction between central neurotransmission and peripheral inflammatory responses in motivation, and they also underscore potential therapeutic approaches that leverage the anti-inflammatory properties of certain treatments.

## 3 Conclusion and future directions

Motivation, and specifically effort-based decision-making, emerges from the coordinated activity of mesolimbic dopamine together with a wider set of neuromodulatory influences, including adenosine, GABA, serotonin, norepinephrine, acetylcholine and metabolic signals. While dopamine remains central to mobilising effort, converging animal and human evidence shows that changes in adenosinergic, cholinergic and pallidal GABAergic pathways, as well as serotonergic receptor–specific mechanisms, can shift cost–benefit evaluations even when reward sensitivity is relatively preserved. Clinically, this may help explain why some patients report intact “liking” yet reduced “willingness to work,” and why dopaminergic interventions alone may leave motivational deficits only partly improved.

In clinical research and practice, constructs such as apathy, anergia and anhedonia are already increasingly recognised as related but dissociable dimensions of motivational dysfunction, each with partly separable neurochemical contributions, with dopaminergic dysfunction more consistently linked to impairments in effort mobilisation and goal-directed behaviour, and other systems contributing to alterations in reward experience or valuation. Building on this trend, future work may benefit from stratifying patients not only by diagnosis but also by predominant motivational profile, which could support more targeted interventions. This perspective could help explain why some individuals respond best to dopaminergic agents, whereas others may benefit more from approaches that engage additional modulatory systems, including adenosine, cholinergic and noradrenergic pathways, or mechanisms linked to stress, inflammation, and metabolism.

Progress will likely depend on establishing reliable methods for translation. Standardised cross-species paradigms that measure variables such as response force, persistence, or preference for high-effort options can make findings more comparable between animal and human studies. When combined with computational models that separate effort cost from reward value, these approaches allow more precise testing of mechanistic hypotheses. In parallel, biologically informed markers, such as MRS-derived metabolites, task-linked fMRI measures of frontostriatal activity, or ligand-based assessments of dopamine and adenosine function, may help demonstrate target engagement and clarify the mechanisms behind early clinical effects.

Overall, the emerging picture is not of a single pathway failure, but of a distributed network in which multiple transmitters and peripheral states shape the willingness to expend effort. For clinicians, an important implication is to view motivational dysfunction as a systems-level problem: dopaminergic treatments may be necessary but are often insufficient on their own, and broader augmentation strategies may be warranted. For researchers, the challenge is to integrate behavioural, computational, and biomarker approaches across species so that descriptive knowledge of neurotransmitter effects can evolve into more principled interventions, while remaining open to revision as new evidence accumulates.

## Figures and Tables

**Table 1:** Representative studies illustrating the neurochemical modulation of effort-based decision-making across species and domains.

System	Study	Species	Domain / Manipulation	Key effect
**Dopamine (DA)**	Salamone et al., (1991)	Animal	Physical/DA depletion / D2 antagonism	Induces low-effort bias; reward sensitivity preserved
	Hosking et al., (2015)	Animal	Physical vs cognitive/DA antagonism	Reduces physical, but not cognitive, effort
	Westbrook et al., (2020)	Human	Cognitive/↑ DA (methylphenidate / sulpiride)	Increases willingness to exert cognitive effort
	Bogdanov et al., (2022)	Human	Cognitive/DA replacement (PD)	Reduces avoidance of high-demand cognitive tasks
	Ang et al., (2023)	Human	Cognitive/MDD-associated DA dysfunction	Reduced willingness to exert cognitive effort
	Treadway et al., (2012)	Human	Physical/Depression (putative DA dysfunction)	Reduced willingness to exert effort
**Adenosine (A2A)**	Farrar et al., (2007)	Animal	Physical/A2A antagonism (MSX-3)	Reverses DA antagonist-induced low-effort bias
	Mingote et al., (2008)	Animal	Physical/A2A activation (agonist)	Reduces effortful responding (DA-like deficit)
**GABA**	Farrar et al., (2008)	Animal	Physical/GABA-A activation (ventral pallidum)	Reduces effortful responding
**Serotonin (5-HT)**	Meyniel et al., (2016)	Human	Physical/SSRI	Reduces perceived effort cost
	Michely et al., (2023)	Human	Cognitive/SRI	Reduces cognitive effort cost
	Nobis et al., (2023)	Human	Physical/SSRI	Bidirectional effects depending on baseline apathy
	Yohn et al., (2016)/Presby et al., (2021)	Animal	Physical/SSRI	Does not restore or suppresses effortful responding (low effort-bias)
**Norepinephrine** **(NE)**	Borderies et al., (2020)	Animal	Physical/↓ NE (clonidine)	Reduces force exertion without affecting reward
	Fitzpatrick et al., (2019)	Animal	Cognitive/attention NE neuron inhibition	Impairs attentional performance under demand
	Varazzani et al., (2015)	Animal	Physical/ Neural recording	NE activity scales with effort; DA encodes reward value
	Soder et al., (2021)	Human	Physical/D-amphetamine (DA/NE)	Increases high-effort choice (mixed catecholamine effect)
**Acetylcholine (ACh)**	Nunes et al., (2013)	Animal	Physical/Muscarinic activation	Reduces effortful responding
	Abdoust et al., 2024	Human	Physical/Muscarinic antagonism	Increases willingness to exert effort
**Stress / Inflammation**	Shafiei et al., (2012)	Animal	Physical/Acute stress	Shifts behaviour toward low-effort choices
	Bryce and Floresco, (2016)	Animal	Physica/l CRH administration	Increases effort cost and reduces vigour
	Dieterich et al., (2021)	Animal	Physical/Chronic stress	Reduces effortful responding
	Draper et al., (2018)	Human	Physical/Endotoxemia (LPS)	Reduces high-effort choice
	Lasselin et al., (2017)	Human	Physical/Endotoxemia (LPS)	Increases high-effort choice (task-dependent effect)
**Metabolic**	Zalachoras et al., (2022)	Human & Animal	Physical ↑ Glutathione (NAc)	Supports sustained effort expenditure
	Wiehler et al., (2022)	Human	Cognitive Cognitive fatigue (glutamate accumulation)	Promotes low-effort preference
**Orexin**	Nasrollahi et al., (2021)	Animal	Physical Orexin-1 receptor activation	Restores effort under DA antagonism

The table summarises key findings from animal and human studies examining how different neurotransmitter systems and related biological signals influence effort-based behaviour. Studies are categorised by neurochemical system and indicate species (animal or human), effort domain (physical or cognitive), and manipulation, as well as the primary effect on effort allocation or decision-making.
